# Understanding risk and protective factors for pain and healthy aging: an intersectional and resilience-based perspective

**DOI:** 10.3389/fpain.2026.1885948

**Published:** 2026-09-02

**Authors:** Joanna M. Hobson, Indonesia A. Jordan, Shinye Kim, Taylor L. Taylor

**Affiliations:** 1 Department of Community Dentistry and Behavioral Sciences, University of Florida, Gainesville, FL, United States; 2 Department of Translational Neuroscience, Wake Forest University School of Medicine, Winston-Salem, NC, United States; 3 Department of Counseling Psychology, University of Wisconsin-Madison, Madison, WI, United States; 4 Department of Family and Community Medicine, University of Alabama at Birmingham, Birmingham, AL, United States

**Keywords:** aging, chronic pain, framework, intersectionality, resilience

## Abstract

Chronic pain (CP) is a major public health concern in the United States, with healthcare costs exceeding $700 billion. Beyond its’ economic burden, CP imposes significant physical, emotional, and functional strain, particularly among populations that are disproportionately affected by pain (i.e., older adults, people with HIV, racial and gender minorities). Despite growing trends in pain research, there remains no consensus on optimal pain management or the mechanisms underlying pain exacerbation. Therefore, this review highlights key risk (e.g., sociodemographic determinants of health, clinical co-morbidities) and protective (e.g., resilience, spirituality, complementary approaches, lifestyle) factors that shape pain experiences and influence trajectories of healthy aging. This manuscript will integrate both risk and resilience promoting factors to inform intervention development, clinical practice, and policy. We discuss intersectionality, defined as the interaction of multiple social identities, as well as its influence on pain and aging trajectories over time. Lastly, we propose a framework that integrates intersectionality and resilience to improve pain outcomes among aging adults.

## 1 Introduction

Chronic pain (CP) is a sensory and emotional experience that is associated with (or resembling) actual or potential tissue damage, and lasts for three months or longer (1). When left unmanaged, people with CP can experience significant mobility limitations, interpersonal and financial stress, and a poorer quality of life (2). This is especially relevant for older adults, and other vulnerable groups that are at an increased risk such as African Americans, people with HIV, women, and people who use substances (2–5). Historically, research has examined risk factors for pain in isolation, such as having poor sleep or expressing symptoms of depression (6–8). However, growing trends have shifted towards understanding how both risk and protective factors work simultaneously to facilitate pain management (9).

Intersectionality refers to the interaction of multiple social identities that shape exposure to health disparities (10, 11). According to prior research, people with greater intersectional identities tend to have poorer health outcomes due to experiencing additive stress daily (12). On the contrary, resilience encompasses psychological, behavioral, and environmental resources that buffer the impact of pain episodes on coping, recovery, sustainability, and growth (13). Although prior reviews examine risk and protective factors in isolation, few adopt an intersectional approach that integrates both barriers and protective factors across aging populations with CP. Thus, the purpose of this perspective is to: 1) expand upon the concepts of intersectionality and resilience by highlighting important risk and protective factors for pain management, and 2) introduce a healthy aging framework that combines these factors to promote optimal pain management among older adults. We argue that pain cannot be adequately treated using siloed risk models, and that integrating intersectionality and resilience into one framework introduces novel, multifactorial intervention strategies.

## 2 Aging with chronic pain: introducing intersectionality

According to the World Health Organization, healthy aging is the process of maintaining functional abilities to enhance well-being in older age, including: meeting daily needs such as household chores, maintaining cognitive and decision-making skills, being physically able to get around, building and maintaining healthy relationships, and continuing to contribute to society (14). Aging healthily is a major concern for older adults, as this group comprises up to 75% of people with a chronic pain condition (15). Specifically, about 21%–75% of older adults are affected by chronic low back pain, and roughly 65% experience osteoarthritis related pain. Following these trends are 35% of older adults that are affected by peripheral neuropathy, and 40% of those who are affected by chronic musculoskeletal pain – which can occur without a traceable injury (15, 16).

Older adults experience two types of pain: primary and secondary conditions (15). Primary pain occurs when pain is the condition, causing significant psychosocial, economical, and physical burden. In secondary conditions, pain is the result of a pre-occurring condition, decreasing quality of life. While older adults primarily report secondary pain, growing literature suggests an increase in the prevalence of CP conditions among this group (15, 16). Importantly, the experience of pain in later life is associated with increased frailty due to altered proprioception, reduced mobility, and a heightened risk for clinical conditions like depression and substance use disorders (8, 15, 17, 18). These overlapping disparities may be due to impaired nociceptive pathways, inflammation, and psychosocial factors (i.e., pain catastrophizing, self-efficacy) amongst others (2, 19–21). Given that pain is complex, an intersectional approach will improve pain management strategies by demonstrating how both risk and protective factors influence pain perception and management.

## 3 Sociodemographic risk factors

### 3.1 Racial disparities

African Americans have historically demonstrated suboptimal pain outcomes, which are heightened as they progress to older age (3, 22). Specifically, African Americans experience greater pain intensity and related disability compared to White individuals (23, 24). A study of 1,925 adults who were aged 50 and older found that African Americans experienced significantly higher pain on average, as well as higher pain impact compared to White and Hispanic individuals (22). In the same study, over half of this group indicated that pain affected their ability to pay for their needs, with interference decreasing as income levels increased. Another study of 1,650 men in a tertiary pain care center found that African Americans reported higher pain severity, related disability, and depressive symptoms compared to other races (25). Moreover, this study found an indirect relationship between African American race and symptoms of depression, psychological distress, post-traumatic stress, and disability through pain severity. Factors such as racial discrimination, financial strain, and provider bias may help explain the relationship between race and pain outcomes (26–29). Moreover, gender and other health-related stigmas often intersect with race, contributing additive burden to pain perception and management in minoritized groups (30–32).

### 3.2 Gender disparities

Gender disparities in pain have also been shown, such that women report greater pain intensity and experimental pain sensitivity compared to men (33). Specifically, women report lower pain tolerance, greater post-operative pain, and higher levels of pain-related psychological distress (i.e., depressive symptoms, fear of movement) compared to men (34, 35). These disparities remain throughout life, often interacting with age to influence pain outcomes. For example, a study of 230 older adults found that 56.4% of women reported a prevalence of chronic pain compared to 29.7% of men (36). In the same study, pain thresholds were lower in women and older adults who had chronic pain compared to those without a CP condition. On the contrary, another study found that although women reported lower heat pain thresholds, men reported greater unpleasantness to mild and moderate pain experiences (37). Moreover, older age and male gender interacted in response to painful stimuli in the insula and right anterior cingulate cortex, highlighting an important region that's involved in the affective-motivational components of pain.

Gender disparities in pain vary by condition, and perceptions of gender identity can often influence pain reports and treatment regimens. For example, a study of 51 African Americans with HIV found that both transgender and cisgender women reported greater pain sensitivity than cisgender men (34). While transgender individuals are often understudied in pain research, growing literature suggests that this group possesses similar hormonal and neurobiological underpinnings as cisgender women, thus mimicking a heightened pain perception and response (33, 34). More research is needed to understand the prevalence and etiology of gender disparities in pain, as well as how age affects these relationships.

### 3.3 Socioeconomic disparities

Socioeconomic status and neighborhood deprivation also predict greater pain severity and disability, with age playing a supporting role in the process (16, 38). People with less socioeconomic resources have reported worse knee pain before and after knee replacement, greater pain interference, increased healthcare usage and reduced life expectancy compared to those that are living in more resourced neighborhoods (39–41). In a study of 188 adults with knee osteoarthritis, higher neighborhood deprivation was associated with greater pain intensity and interference, and higher income and education were associated with lower pain outcomes (40). For this study, older age was associated with lower pain ratings compared to younger adults. Other studies have found that older age heightens vulnerability for pain due to environmental exposures, mobility issues, and reliance on community resources (42). Taken together, these studies highlight the intersection of socioeconomic status, older age, and an increased vulnerability to pain and disability.

## 4 Clinical risk factors

### 4.1 Human immunosuppressive virus (HIV)

In addition to sociodemographic risk, clinical risk factors like human immunosuppressive virus (HIV) often intersect with aging to influence pain trajectories. To elaborate, roughly 85% of people with HIV have a pain condition, and these conditions are associated with missed healthcare visits, reduced treatment adherence, functional limitations, and lower quality of life (43, 44). Historically, pain was neuropathic in nature due to the toxic effects of early antiretroviral medications (i.e., nucleoside analog reverse-transcriptase inhibitors) (44). However, treatment has advanced over time, thus expanding the lifespan of people with HIV and shifting symptoms from neuropathic to non-neuropathic (e.g., musculoskeletal) pain (45).

Older adults comprise of over 50% of the HIV and pain population, which shifts attention from managing a life-threatening disease to treating pain symptoms in later life (46). Moreover, this group often experiences cumulative stress including racial, gender, and health-related stigma, financial insecurity, criminalization, and intimate partner violence compared to people without HIV (47–50). Consistent and additive experiences of stress increase allostatic load, which heightens susceptibility to chronic pain and accelerated aging (51, 52). Nonetheless, future research should aim to disentangle the neurobiological and psychological predictors of pain and aging in people with HIV.

### 4.2 Insomnia

Insomnia is also highly prevalent in people with CP, affecting 40%–50% in older adults compared to the general population (53). Studies show a bi-directional relationship between sleep and pain, such that patients with CP frequently report insomnia symptoms, while severe cases of insomnia have been linked to hyperalgesia, and the development of pain (7). Notably, growing longitudinal evidence suggests that poor sleep is a stronger predictor of pain outcomes than pain is of subsequent sleep disturbance (7). Specifically, frequent sleep disturbances increase the likelihood of developing CP, heightening existing pain symptoms (i.e., tension headaches, fibromyalgia, rheumatoid arthritis), and decreasing quality of life. Moreover, mechanisms underlying this association have also been implicated, such as lifestyle and psychological states, occupational and environmental stressors, comorbid conditions (i.e., depression, sleep apnea), neuroinflammation, and HPA axis dysregulation (54).

### 4.3 Substance use

Substance use co-occurs with pain conditions, such that at least 40% of people with CP meet criteria for a substance use disorder, and 50% of patients with a substance use disorder report CP (55, 56). This finding is alarming, given the rising rates of opioid overdoses and risk among pain populations as they age (57, 58). Treating pain in older adults has become increasingly difficult due to the risk of polypharmacy, drug-drug interactions, and comorbid conditions that sometimes counteract pain treatment (46, 59, 60). Moreover, studies have shown that approximately 50% of older adults who misuse opioids have obtained them through their physician (60). The ubiquitous nature of pain and substance use suggests that this relationship be studied more extensively in older adults, especially for those who identify with one or more minoritized groups (i.e., African American, older adult, chronic pain, HIV). More research is needed to understand the intersection between sociodemographic and clinical risk factors, pain outcomes, and substance use in older adults to facilitate tailored pain management interventions.

### 4.4 Post-traumatic stress

Post traumatic stress disorder (PTSD) is another disparate yet understudied risk factor for accelerated aging in people with CP (61). PTSD occurs when exposure to actual or threatened harm results in persistent symptoms for more than one month (i.e., intrusion, avoidance, negative mood, hyperarousal) that cause significant distress or functional impairment (62). At least 30% of people with CP have a diagnosis of PTSD, and this number varies in subpopulations such as people with HIV (33%), and people who use opioids (∼95%) (63–65). Older adults who experienced chronic traumatic events develop a ‘triple diagnosis’ of pain, post-traumatic stress, and a mental health condition such as a substance use disorder (65–67). This triple diagnosis may interrupt treatment and medication adherence, thus leaving older adults at risk for further complications (i.e., frailty, mortality risk, illicit use). Some of the most common factors that influence pain and PTSD are depression and pain catastrophizing, which often coincide with maladaptive coping behaviors that arise following stressful events (68–72). Given the potential for increased disability, healthcare costs, and wear and tear on the brain and body, it's imperative to recognize PTSD as a clinical risk factor for pain in older adults (73–75).

Taken together, sociodemographic and clinical risk factors sit at the intersection of pain and aging. As previously discussed, pain is disparately prevalent and heightened in those with greater socioeconomic risk. African Americans, sexual minorities, people with lower socioeconomic status, and those with clinical conditions (i.e., HIV, insomnia, substance use, PTSD) experience heightened pain, psychological distress, and economic burden compared to the general population. This burden is heightened in older adults, introducing a slew of treatment issues, further exacerbating pain, and accelerating the aging process. One of the major issues for inconsistency in pain treatment is designing measures of intersectionality, thus capturing multiple conditions, and etiologies, in one space (10). Thus, the need for tailored interventions and affordable, translational treatments for pain in older adults remains substantial. The following sections will incorporate alternative approaches to protect against, and/or manage CP in minoritized groups.

## 5 Building resilience to manage pain: protective practices

While much research has focused on risk reduction, a growing body of literature highlights the central role of protective factors in pain adaptation, recovery, and long-term self-management (9, 13, 76). Resilience is a growing area of interest, and is defined as the ability to adapt to, manage, or recover from stressful situations (e.g., a pain episode) (13). The three main outcomes of resilience are recovery, sustainability, and growth (13). Recovery refers to a person's ability to return to homeostasis physiologically or regroup mentally after cognitive and affective disturbances. Sustainability is the ability to maintain daily goals, engage socially, and maintain prior activities that boost self-esteem and positive emotions. Lastly, growth refers to benefit finding or using stressful experiences as a chance to learn from them and adapt (13). For older adults with CP, these 3 facets depend on their intrinsic resources and external environments, such as having optimism and a strong social support system (77–79).

For older adults, adapting to a pain episode depends on three main factors: intrinsic resources, vulnerability traits, and sociodemographic characteristics that intersect to shape pain outcomes (13). According to Sturgeon et al, pain-specific resilience outcomes (recovery, sustainability, growth) depend on individual coping strategies, which are informed by both protective and vulnerability mechanisms (13). Personal resilience resources such as optimism, meaning making, pain self-efficacy, social support, and positive emotions (i.e., positive affect, hope, grit) are associated with lower pain intensity and catastrophizing, shorter recovery time following knee replacement, and decreased depressive symptoms that often accompany pain (13). On the contrary, vulnerability traits like re-occurring depression, internalized stigma, and adverse childhood/adult experiences can influence ‘vulnerability’ or maladaptive coping mechanisms, such as pain catastrophizing, negative affect, and social isolation (13, 80–82). Importantly, intrinsic and external resources depend on the social, economic, and cultural environment that a person is in (13). Thus, the following sections will describe protective factors that may improve pain management, recognizing that some factors will be less accessible or function differently in structurally marginalized groups (9, 83, 84).

### 5.1 Spirituality – prayer for pain relief

Spirituality involves having or searching for an individual purpose in life that is bigger than oneself or connected to a higher power (85, 86). For some, spirituality serves as a source of hope when faced with challenging situations. Several studies suggest that prayer can function as a pain-coping strategy and may be associated with improved physical functioning, pain tolerance, and the use of adaptive coping strategies within certain cultural contexts (87–89). To elaborate, a study of 70 patients undergoing appendicectomies found that listening to prayer predicted lower post-operative pain and heart rates as compared to music and controls (90). Another study found that African Americans were more likely to practice prayer as a coping strategy, and that active prayer was associated with a higher pain tolerance compared to those with passive praying strategies, or no prayer (87). On the contrary, other studies have found that prayer and spirituality were associated with worse pain outcomes, such as functional impairment, disability, and greater pain severity (89, 91).

Discrepancies in pain outcomes can be partially explained by cultural variability, and individual perceptions of the meaning of pain. Some individuals may perceive a pain flare up as a punishment, or an attack that should be prayed away (86). Others may conceptualize their pain experience as something that can be healed actively, thus working towards managing their pain (86). Factors like positive reappraisal and pain catastrophizing serve as buffers in this relationship (88). Nonetheless, prayer serves as source of resilience, while other practices (i.e., yoga, music, meditation) are increasingly used for pain management and intervention.

### 5.2 Complementary medicine – yoga and mediation

Complementary approaches such as yoga and mindfulness-meditation have been effective in managing pain symptoms (92, 93). Briefly, yoga is an ancient mind-body practice that combines physical movement with mental focus (93). The primary purpose of yoga is to assist in muscle strength, flexibility, and overall spiritual balance. However, recent literature suggests that yoga is a cost-efficient treatment for chronic low back pain, fibromyalgia, neck pain, and headaches – common symptoms and conditions that occur in older adults. A systematic review of 27 studies (*n* = 2,702) found that compared to controls, yoga was associated with short and long-term improvements in pain intensity and related disability, as well as mental and physical functioning in people with chronic low back pain (92). Other studies have found that yoga improves psychological symptoms such as emotional distress, anxiety and depression, which often coincide with pain symptoms (92, 94). What remains unclear, however, is the effects of yoga on resilience traits and resources (i.e., hope, pain self-efficacy), or whether this practice works in minoritized groups.

Mindfulness meditation is a self-regulatory practice that involves paying attention to your body's sensations, internal dialogue, and the external environment, without ruminating on a single thought or feeling (95). Consequently, emotional distress should be reduced, thus lessening the severity and perception of pain. Studies show that spending 3–4 days practicing mindful meditation has significant effects on pain intensity and pain unpleasantness (96–98). Also, practicing meditation for 2-weeks following a 20-minute training session was shown to increase pain thresholds while decreasing pain intensity in healthy adults (99). Mindfulness meditation also increases feelings of hope and meaning making, which are key factors for promoting resilience, or overcoming adversity. The caveat to using yoga and mindfulness-meditation practices is that they’re usually taught, which leaves the responsibility on the patient, and involves limitations such as accessibility, cultural relevance, and health literacy which could be considered when implementing these approaches in minoritized older adults (100).

### 5.3 Positive activity interventions

Resilience traits can also be built through promoting activities that increase positive emotions (101–103). For example, doing activities that increase feelings of positive affect, pain acceptance, hopeful thinking, and pain self-efficacy has strong effects on pain intensity, disability, and emotional function in older adults (102, 104). Moreover, engaging in random acts of kindness, spending time with friends, exercising regularly, and listening to music that increases positive emotions has improved pain interference, global functioning, and quality of life in African American older adults (103). Importantly, positive activity interventions involve multiple components but share a common mechanism of recognizing both risk and resilience factors that affect pain perception. Originally developed to reduce pain, positive activity interventions decrease stress and promote emotion regulation, a positive mood, and feelings of self-efficacy – all of which are major components for coping with acute and chronic pain. Future research should establish the feasibility of positive psychology interventions in older adults and other risk groups that may not adhere to traditional pain treatment.

### 5.4 Daily practices: music and expressive writing

Lastly, recent work demonstrates that practices like listening to music and expressive writing have specific effects on pain, traumatic stress symptoms, and substance use in vulnerable populations (105–108). Studies show a direct relationship between music and pain, such that people with HIV, pain, and opioid use report lower levels of negative affect and pain catastrophizing after listening to music for 10-minutes (105). Also, adults in the emergency department for pain symptoms reported significant reductions in pain and anxiety following a smartphone-based music intervention (109). Music activates some of the same regions in the central nervous system that are responsible for the cognitive-affective and motivational components of pain (105). However, music-based interventions are scarce among older adults, as well as other racial, sexual, and clinical risk populations. Given the potential for music to decrease pain, avoidant coping, and increase positive emotions, future research should consider it as a cost effective, translational, and feasible intervention for pain management.

Expressive writing is a form of emotional awareness and expression therapy that involves writing for 10–15 min about a stressful experience without judgement, and without worrying about grammar (110). Studies show that expressive writing helps to relieve pain by relaxing the mind and separating the person from the experience (111). Moreover, expressive writing helps to release pint-up emotions, while also increasing feelings of hopefulness and a positive mood in vulnerable populations (112). Studies using expressive writing in people with HIV found an increase in medication adherence, social support, self-efficacy, and a decrease in symptoms of depression, anxiety, and insomnia (106, 113). Importantly, these factors all intersect to facilitate pain in older adults and should be explored further in groups who experience significant and unique stress.

Taken together, positive activity interventions, spiritual practices, mind-body approaches, and daily practices such as listening to music and journaling offer novel approaches to pain management interventions. This is especially relevant in older adults that comprise multiple intersectional identities, and are at risk for physical, psychological, and socioeconomic burden.

## 6 Discussion: developing a framework

The purpose of this review was to identify risk and protective factors for pain and healthy aging using an intersectional and resilience-based approach. Based upon our review, sociodemographic and clinical risk groups intersect with aging to influence pain outcomes. Also, protective factors such as spirituality, yoga, mindfulness meditation, and daily practices (i.e., listening to music, journaling) help build resilience, which has proven to reduce the burden of pain on older adults. Although prior research demonstrates the effects of risk and resilience on pain, scarce literature has combined these factors to inform future pain interventions and treatment. We argue that analyzing pain from an intersectional and resilient perspective will contribute to optimal pain management by accounting for both positive and negative mechanisms.

**Figure 1:**
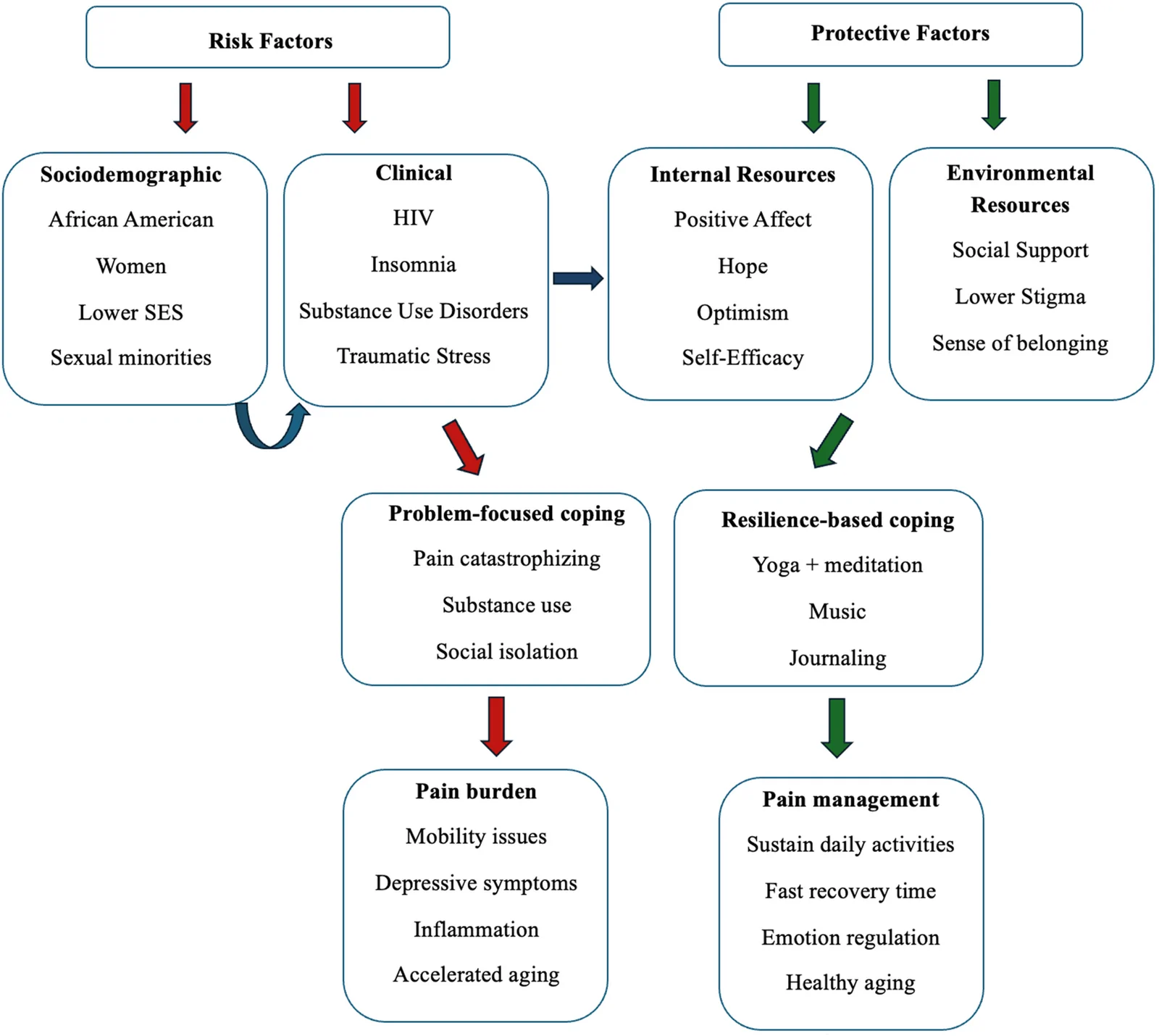
Intersectional risk and resilience framework informed by Sturgeon et al. Sociodemographic and clinical risk groups intersect to inform coping strategies that ultimately affect the burden of pain. However, internal and environmental resources can be altered to promote resilience-based coping, thus changing pain burden to pain management through sustainability, recovery, regulation, and growth (i.e., healthy aging).

We propose a framework (Figure 1) that integrates measures of risk and resilience to inform pain management strategies. Vulnerable populations such as African Americans, women, sexual minorities, and people with lower socioeconomic status experience greater pain compared to other groups (22, 23, 37, 38). Moreover, pain intersects with clinical conditions such as HIV, insomnia, substance use disorders, and post-traumatic stress (45, 54, 56, 114). Oftentimes, sociodemographic and clinical characteristics co-exist, and manifest as additive stress, greater pain, and less cognitive resources to respond to pain episodes (115). For example, a study of 97 people with HIV and pain found that greater intersectional stigma predicted greater depression, insomnia, and pain severity compared to those with lower or moderate levels of stigma (31). Another study found that people with HIV and pain who used opioids had greater depressive and insomnia symptoms compared to those who didn't use opioids (116). Importantly, both studies consisted of predominantly African American men who sleep with men and lived below the poverty level – thus highlighting overlapping risk factors that could be captured in future research. Lastly, sociodemographic disparities in pain share similar trends in other conditions, such as insomnia, PTSD, HIV, and substance use disorders (55, 69, 117). Future research should consider developing a standard measure of intersectionality, comprising both demographic and clinical risk groups for pain outcomes.

Implementing protective factors to promote resilience offers promising benefits to pain management for older adults (13). Briefly, internal resources such as positive affect, hope, optimism, and pain self-efficacy increases the likelihood of adaptive coping (i.e., spending time with friends, journaling, exercise) (13). In turn, older adults who experience pain will be able to sustain daily activities, recover from the intensity of pain, and grow from that experience. However, internal resilience resources often depend on measures of intersectional risk, as well as the external environment that a person has access to (13). If a person has more vulnerability traits (i.e., adverse childhood experiences, reoccurring depression) and a less resourceful environment (i.e., less social support, financial strain), the capacity to cope effectively with pain may be limited (13). However, resilience-based interventions assist in re-building psychological traits (i.e., meaning making), sharing resources, and ultimately feeling confident in the ability to manage pain and related stressors.

### 6.1 Strengths and limitations

Our review has both strengths and limitations. This work is novel because we highlight that treating pain is not a siloed approach, and that sociodemographic and clinical factors often intersect to influence pain outcomes. Moreover, we present protective factors that are understudied in minoritized groups, and could further pain treatment by developing culturally tailored, accessible interventions. Combining intersectional and resilience-promoting factors aligns with the whole person health model, thus shifting towards treating the whole person and not individual organs or symptoms (118). Nonetheless, there's no consensus on measuring intersectionality, and future work should incorporate a standardized measure to address groups that may be missed (10). Lastly, some of the clinical risk groups are often excluded from pain research, thus limiting the ability to capture additional variance.

## 7 Conclusions

Based upon this framework, we suggest that future research considers the multifactorial layers of pain patients (i.e., clinical and sociodemographic risk), as well as non-pharmacological treatments (i.e., positive activities, yoga, music, journaling) that can be translated into daily life. Advancing this field will require integrating intersectionality measures into study design, prioritizing resilience-based mechanisms as modifiable targets, and developing culturally responsive, accessible interventions for aging populations with CP.
